# Can mass drug administration of moxidectin accelerate onchocerciasis elimination in Africa?

**DOI:** 10.1098/rstb.2022.0277

**Published:** 2023-10-09

**Authors:** Klodeta Kura, Philip Milton, Jonathan I. D. Hamley, Martin Walker, Didier K. Bakajika, Eric M. Kanza, Nicholas O. Opoku, Hayford Howard, Maurice M. Nigo, Sampson Asare, George Olipoh, Simon K. Attah, Germain L. Mambandu, Kambale Kasonia Kennedy, Kambale Kataliko, Mupenzi Mumbere, Christine M. Halleux, Adrian Hopkins, Annette C. Kuesel, Sally Kinrade, Maria-Gloria Basáñez

**Affiliations:** ^1^ London Centre for Neglected Tropical Disease Research, Department of Infectious Disease Epidemiology, School of Public Health, Imperial College London, London W2 1PG, UK; ^2^ MRC Centre for Global Infectious Disease Analysis, Department of Infectious Disease Epidemiology, School of Public Health, Imperial College London, London W2 1PG, UK; ^3^ Department of Pathobiology and Population Sciences, Royal Veterinary College, Hatfield AL9 7TA, UK; ^4^ Expanded Special Project for Elimination of Neglected Tropical Diseases (ESPEN), African Regional Office of the World Health Organization (WHO/AFRO/ESPEN), Brazzaville, Democratic Republic of Congo; ^5^ Programme Nationale de Lutte contre les Maladies Tropicales Négligées à Chimiothérapie Préventive (PNLMTN-CTP), Ministère de la Santé Publique, Kinshasa, Democratic Republic of the Congo; ^6^ Department of Epidemiology and Biostatistics, School of Public Health, University of Health and Allied Sciences, Hohoe, Ghana; ^7^ Liberia Institute for Biomedical Research (LIBR), Monrovia, Liberia; ^8^ Institut Supérieur des Techniques Médicales de Nyankunde, Bunia, Democratic Republic of the Congo; ^9^ GlycoScience Research, Brookings, SD 57006, USA; ^10^ Precious Minerals Marketing Company, National Assay Centre, Technical Department, Diamond House, Accra, GA-143-2548, Ghana; ^11^ Department of Medical Microbiology, University of Ghana Medical School, College of Health Sciences, Accra, Ghana; ^12^ Inspection Provinciale de la Santé de la Tshopo, Kisangani, Democratic Republic of the Congo; ^13^ Department of Clinical Research, London School of Hygiene and Tropical Medicine, London WC1E 7HT, UK; ^14^ Centre de Santé CECA 20 de Mabakanga, Beni, Nord Kivu, Democratic Republic of the Congo; ^15^ Medicines Development for Global Health, 18 Kavanagh Street, Southbank, Victoria 3006, Australia; ^16^ UNICEF/UNDP/World Bank/WHO Special Programme for Research and Training in Tropical Diseases (TDR), World Health Organization, 1211 Geneva 27, Switzerland; ^17^ Neglected and Disabling Diseases of Poverty Consultant, Gravesend, Kent DA11 OSL, UK

**Keywords:** onchocerciasis, moxidectin, ivermectin, mass drug administration, MDA, modelling

## Abstract

Epidemiological and modelling studies suggest that elimination of *Onchocerca volvulus* transmission (EoT) throughout Africa may not be achievable with annual mass drug administration (MDA) of ivermectin alone, particularly in areas of high endemicity and vector density. Single-dose Phase II and III clinical trials demonstrated moxidectin's superiority over ivermectin for prolonged clearance of *O. volvulus* microfilariae. We used the stochastic, individual-based EPIONCHO-IBM model to compare the probabilities of reaching EoT between ivermectin and moxidectin MDA for a range of endemicity levels (30 to 70% baseline microfilarial prevalence), treatment frequencies (annual and biannual) and therapeutic coverage/adherence values (65 and 80% of total population, with, respectively, 5 and 1% of systematic non-adherence). EPIONCHO-IBM's projections indicate that biannual (six-monthly) moxidectin MDA can reduce by half the number of years necessary to achieve EoT in mesoendemic areas and might be the only strategy that can achieve EoT in hyperendemic areas. Data needed to improve modelling projections include (i) the effect of repeated annual and biannual moxidectin treatment; (ii) inter- and intra-individual variation in response to successive treatments with moxidectin or ivermectin; (iii) the effect of moxidectin and ivermectin treatment on L3 development into adult worms; and (iv) patterns of adherence to moxidectin and ivermectin MDA.

This article is part of the theme issue ‘Challenges in the fight against neglected tropical diseases: a decade from the London Declaration on NTDs’.

## 1. Introduction

Onchocerciasis (river blindness) is a vector-borne neglected tropical disease (NTD) caused by the filarial nematode *Onchocerca volvulus*. It affects millions of people in several endemic regions, with greater than 99% living in sub-Saharan Africa. The vectors are blackflies of the genus *Simulium*, and in Africa the main vectors belong to the *Simulium damnosum sensu lato* (s.l.) species complex. Annual mass drug administration (MDA) of ivermectin for those aged greater than or equal to 5 years was introduced in the early 1990s. For countries under the umbrella of the African Programme for Onchocerciasis Control (APOC, 1995–2015), MDA has been delivered by community-directed treatment with ivermectin (CDTI), mostly annually (aCDTI) [1].

Ivermectin's effects on *O. volvulus* are deemed to be primarily threefold: (i) clearance of skin microfilariae (mf) (so-called microfilaricidal effect); (ii) temporary disruption of microfilarial production by adult female worms (so-called embryostatic effect); and (iii) permanent sterilization of female worms (so-called cumulative, irreversible, effect on female worm microfilarial productivity, referred to subsequently as ‘sterilizing effect’) [2,3]. Other effects that have been proposed include impacts on female insemination rates [4], adult worm viability, shortening its lifespan (macrofilaricidal effect) [5] and incoming (L3–L5) stages (prophylactic effect) [6]. This paper uses the first three effects to model the impact of ivermectin or moxidectin MDA on *O. volvulus* populations.

The World Health Organization (WHO) 2021–2030 roadmap for NTDs targets elimination (permanent interruption) of *O. volvulus* transmission (EoT) and has proposed that this be verified in 12 (31%) countries by 2030 [7]. EoT is deemed to have been achieved when infectivity in blackfly samples (by polymerase chain reaction (PCR) amplification of the *Onchocerca*-specific repeat O-150 sequence) is less than 0.05% (at the upper 95% confidence limit, CL) and IgG4 antibody seropositivity to the *O. volvulus* Ov16 antigen is less than 0.1% (also at the upper 95% CL) in children aged less than 10 years [8]. EoT has been verified by WHO in four out of the six formerly endemic countries in the Americas [9,10] and has been reported (according to the criteria described above) in some foci in Africa [11–14]. However, it is highly unlikely that aCDTI alone can achieve the 2030 targets in all foci [15]. Four major impediments include: (i) endemic communities that have not yet received treatment or have only started MDA recently; (ii) communities under treatment in areas with high vector biting rates, insufficient treatment coverage (proportion of the population taking the drug during a given treatment round) and/or high levels of systematic non-adherence (proportion of the population never taking the drug); (iii) communities with so-called sub-optimal responses to (the embryostatic effect of) ivermectin [16,17] and (iv) areas co-endemic with loiasis (another filarial infection, caused by *Loa loa* and transmitted by tabanid vectors) owing to the risk of severe adverse reactions to ivermectin in individuals with high levels of *L. loa* microfilaraemia [18].

Communities that have not yet received treatment or have only started MDA recently are primarily in hypoendemic areas. This is due to the fact that until 2011/2012 APOC's objective was elimination of onchocerciasis as a public health problem (EPHP) [1] and aCDTI prioritized meso- and hyperendemic areas (characterized, respectively, by a prevalence of palpable onchocercal nodules—where a fraction of the adult worms resides—of 20–39%, with a microfilarial prevalence of 40–59%, and a nodule prevalence greater than or equal to 40%, with microfilarial prevalence greater than or equal to 60%) [19]. Communities in hypoendemic areas were included in CDTI when they were part of a health system administrative unit that included meso- and hyperendemic areas, and in MDA with ivermectin and albendazole if they were co-endemic for lymphatic filariasis [20]. To achieve sustainable EoT, hypoendemic areas need to be included in interventions since they could represent a potential parasite reservoir, impeding elimination [21].

Most, but not all, mesoendemic and hyperendemic communities in non-loiasis co-endemic areas have received aCDTI for several years. Many communities are broadly on track [22], but transmission persists in others despite prolonged treatment [23,24]. In highly endemic communities, transmission from humans to vectors may occur between treatment rounds even when CDTI's coverage is high [25]. This is driven by factors that also apply to other areas, including: (i) ivermectin does not result in the death of all mf in all infected people treated [2,26]; (ii) the susceptibility of *O. volvulus* to ivermectin's embryostatic effect varies widely, resulting in repopulation of the skin with mf that can be ingested by vectors following ivermectin treatment [27,28]; (iii) not all eligible individuals take treatment in each treatment round [29]; (iv) timing of ivermectin treatment relative to transmission seasonality may not be optimal [20]. Studies across Africa have found that the proportion of individuals that have never or very infrequently taken ivermectin can exceed 10% [29–33].

Besides optimization of aCDTI implementation (increasing coverage, reducing systematic non-adherence and improving treatment timing), alternative treatment strategies (ATSs) have been proposed to accelerate progress towards EoT [20,34]. Increasing ivermectin treatment frequency to biannual (bCDTI) is predicted to shorten time to elimination [35], but few countries have adopted this strategy owing to lack of resources, programmatic costs and/or feasibility and logistics of access to various communities if they are to be reached twice yearly [36]. Complementary vector control has also been proposed. For example, the community-directed vector control method known as ‘slash and clear’, where feasible to implement, can potentially accelerate elimination by decreasing the blackfly population density and biting rates [20,37]. ‘Test and not treat’ strategies to exclude *L. loa*-infected individuals with heavy microfilaraemia and thus at risk of severe adverse reactions to ivermectin have been trialled successfully [38].

ATSs also include new drugs [34,39,40]. Moxidectin, a milbemycin endectocide hailing from the veterinary field [41], was approved in 2018 by the US FDA for the treatment of human onchocerciasis in those aged greater than or equal to 12 years [42]. Phase II [26] and Phase III [27,28] clinical trials have demonstrated that moxidectin is significantly more efficacious than ivermectin in reducing skin microfilarial loads in more people, to a greater extent and for longer periods, with a comparable safety profile. A review of moxidectin for the treatment of human onchocerciasis can be found in Milton *et al*. [40]. An overview of available data and ongoing studies was provided for discussion of the potential of moxidectin for onchocerciasis elimination at the 2022 meeting of the Coalition for Operational Research on NTDs (COR-NTD) [43].

In this paper, we expand our previous deterministic modelling study [44] by using our individual-based, stochastic transmission model (EPIONCHO-IBM), developed at Imperial College London [45], to project the epidemiological impact of MDA with ivermectin or moxidectin. Particularly, we generate projections on elimination probabilities and estimate the number of years of treatment needed to achieve EoT under different assumptions of baseline endemicity, treatment coverage, systematic non-adherence and treatment frequency with a focus on treatment-naive settings. While moxidectin is not yet approved for treatment of children under the age of 12, we have assumed that MDA with either drug is delivered to all aged greater than or equal to 5 years to facilitate comparison between CDTI or community-directed treatment with moxidectin (CDTM). We consider this is justified given that the trial to identify a moxidectin dose for 4- to 11-year-old children that achieves exposures comparable to those in adults receiving an 8 mg dose has been completed [46], and preparation for expanding the safety database for 4- to 11-year olds is under way.

## 2. Methods

### (a) EPIONCHO-IBM

EPIONCHO-IBM is a stochastic individual-based model that extends our previous deterministic, population-based, EPIONCHO model [35,47,48] and comprises a sub-model for the parasite's life cycle stages in humans and another for the stages in the vector. The human sub-model assumes a closed population of 440 individuals, and tracks, within individual hosts, the number of *O. volvulus* adult worms (macrofilariae)—categorized by sex (male and female worms), age and reproductive status—and of skin mf. The model accounts for age- and sex-dependent exposure of humans to blackfly bites and incorporates individual-level variation in exposure to drive the characteristic aggregated distribution of parasites among humans. The life cycle stages in the vector are modelled deterministically, using delay differential equations to capture the mean numbers of L1, L2 and L3 larvae per blackfly. A detailed mathematical description can be found in Hamley *et al*. [45].

### (b) Drug effects

Both ivermectin and moxidectin are modelled as having microfilaricidal, embryostatic and irreversible female worm-sterilizing effects [2,3,44]. For both drugs, the same set of equations is used to model the microfilaricidal and embryostatic effects as presented in Basáñez *et al*. [2], but with different parameter values to capture the microfilarial dynamics that ensue following treatment with each drug. The parameters for ivermectin had been estimated by fitting such equations to the temporal trends (up to 24 months following treatment) of microfilarial load from 15 studies of single-dose (150 µg kg^−1^) ivermectin, with estimation of the embryostatic function supported by data on the proportion of female worms containing live mf (up to 20 months) from three studies examining ivermectin's effect on macrofilariae [2]. The moxidectin parameters were estimated based on the skin microfilarial densities in the Phase II moxidectin clinical trial pre-treatment as well as at 8 days and 1, 2, 3, 6, 12 and 18 months post-treatment with a single dose of 8 mg moxidectin [44]. For both drugs, the proportion of female worms sterilized after each treatment round was assumed to be 0.345 (35%), based on modelling presented in Plaisier *et al*. [3] using longitudinal data on mf counts in adults receiving one or five annual ivermectin treatments, obtained in Ghana [49,50] (table S1). A sensitivity analysis was also conducted (see below).

Electronic supplementary material, figure S1 presents the post-treatment microfilarial dynamics following a single standard (150 µg kg^−1^) dose of ivermectin or an 8 mg dose of moxidectin in the Phase II and Phase III trials [26,27]. The equations, parameter values for ivermectin and moxidectin, and the approach for parameter validation are described in electronic supplementary material, text S1 ('Modelling ivermectin and moxidectin treatment effects and dynamics') and electronic supplementary material, text S2 ('Validation of EPIONCHO-IBM to Phase II and Phase III clinical trial data').

### (c) Overview of scenarios simulated

To investigate the impact of CDTI or CDTM on achieving EoT, we considered primarily treatment-naive hypoendemic (30% mf prevalence), mesoendemic (50% mf prevalence) and hyperendemic (70% mf prevalence) settings in the population aged greater than or equal to 5 years, at baseline.

To obtain these mf prevalences, we sampled across annual biting rates (ABRs) (electronic supplementary material, text S3.1 and figure S2). Therefore, each endemicity scenario is described by a range of biting rates, reflecting inherent uncertainty in transmission conditions in different foci (i.e. foci are classified as hypo-, meso- or hyperendemic, but their exact transmission conditions are uncertain). The results obtained with this approach, for treatment-naive scenarios, are presented in the main text. An alternative method for sampling biting rates is presented in the electronic supplementary material, text S3.2 ('Alternative method for sampling biting rates to represent endemicity categories') and electronic supplementary material, figure S3. This alternative method was used to examine the range of ABRs in which EoT can be achieved in treatment-naive hyperendemic areas (electronic supplementary material, figure S4) and the impact of CDTI or CDTM in meso- and hyperendemic areas with 20 years of prior aCDTI (electronic supplementary material, figures S5 and S6).

Coverage and adherence values assumed include ‘minimal coverage’ (65% of the total population, the target treatment coverage for EPHP [51,52], with 5% systematic non-adherence) and ‘enhanced coverage’ (80% total population coverage, 1% systematic non-adherence), following other modelling studies [35,45,47]. Minimal and enhanced coverage correspond to approximately equal to 80% and approximately equal to 100% of the eligible population taking the drug. Individuals younger than 5 years of age and pregnant women (as well as those breastfeeding a baby younger than one week) are not part of the eligible population [53]. For both coverage scenarios, annual and biannual (six-monthly) MDA with ivermectin (aCDTI, bCDTI) or moxidectin (aCDTM, bCDTM) were modelled, for a maximum programme duration of 40 years.

The epidemiological impact of these strategies was assessed by calculating the probability of elimination 50 years after the last treatment round (measured after every 2 years of treatment) as the proportion of 500 simulations that resulted in elimination, defining elimination as mf prevalence being equal to 0%. Comparisons of programme durations (number of years required) refer to achieving a 90% elimination probability.

### (d) Sensitivity analysis

Given the uncertainty around the cumulative (sterilizing) effect of repeated moxidectin and ivermectin exposure of adult worms on their microfilarial production (there are as yet no data on repeat moxidectin treatment, and the data for repeat ivermectin only comprised a sample of 114 adults), a sensitivity analysis was conducted to investigate the impact of varying the magnitude of the sterilizing effect. Given moxidectin's longer half-life [40] and more prolonged effect on microfilaridermia after a single dose [26–28], it is plausible that the cumulative effect of moxidectin may be greater than that of ivermectin, but currently there are no data with which to scrutinize this conjecture. We decreased the cumulative effect due to ivermectin by half (to 17.5%) while keeping that of moxidectin at 35%, per dose to explore the impact of differences in the cumulative sterilizing effect of moxidectin and ivermectin given that studies other than [3] do not support a 35% cumulative effect of ivermectin [44]. We explored treatment-naive hypoendemic (30% mf prevalence), mesoendemic (50% mf prevalence) and hyperendemic (70% mf prevalence) settings in the population aged greater than or equal to 5 years at baseline, with minimal coverage (electronic supplementary material, text S4 and figures S7–S9).

Throughout the analysis, we followed the five principles of the NTD Modelling Consortium regarding Policy-Relevant Items for Reporting Models in Epidemiology of Neglected Tropical Diseases (PRIME-NTD), for good practice in NTD modelling (electronic supplementary material, text S5 and table S2).

## 3. Results

**Figure 1.**
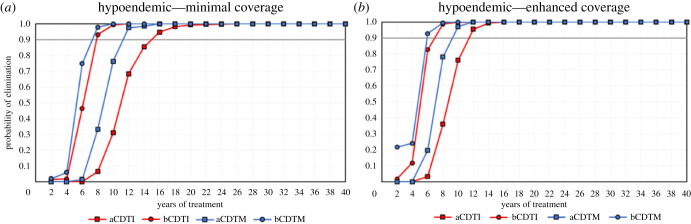
The probability of elimination (proportion of 500 model runs that achieve elimination, defined as 0% mf prevalence 50 years after the last treatment round) for previously untreated hypoendemic scenarios (baseline 30% mf prevalence) versus number of years of annual (squares) or biannual (six-monthly) (circles) community-directed treatment with ivermectin (CDTI, red markers and lines) or moxidectin (CDTM, blue markers and lines) assuming: (*a*) minimal coverage (65% treatment coverage of total population and 5% systematic non-adherence); (*b*) enhanced coverage (80% treatment coverage of total population and 1% systematic non-adherence). CDTI and CDTM are preceded by the letter ‘a’ for annual or ‘b’ for biannual treatment. The grey horizontal line indicates 90% elimination probability. (Online version in colour.)

**Figure 2.**
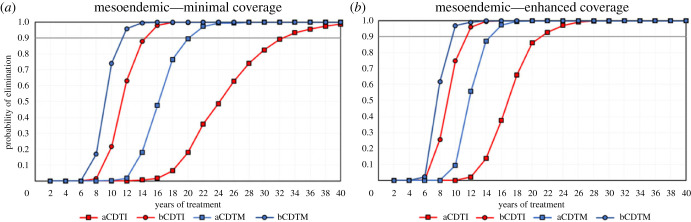
The probability of elimination (proportion of 500 model runs that achieve elimination, defined as 0% mf prevalence 50 years after the last treatment round) for previously untreated mesoendemic scenarios (baseline 50% mf prevalence) versus number of years of annual (squares) or biannual (six-monthly) (circles) community-directed treatment with ivermectin (CDTI, red markers and lines) or moxidectin (CDTM, blue markers and lines) assuming: (*a*) minimal coverage (65% treatment coverage of total population and 5% systematic non-adherence); (*b*) enhanced coverage (80% treatment coverage of total population and 1% systematic non-adherence). CDTI and CDTM are preceded by the letter ‘a’ for annual or ‘b’ for biannual treatment. The grey horizontal line indicates 90% elimination probability. (Online version in colour.)

**Figure 3.**
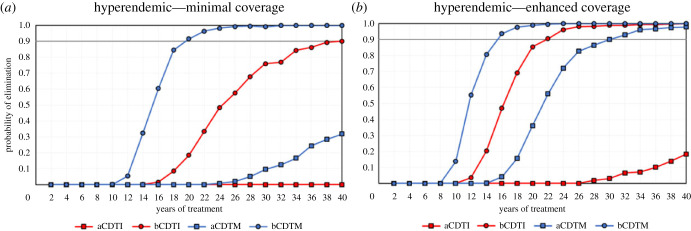
The probability of elimination (proportion of 500 model runs that achieve elimination, defined as 0% mf prevalence 50 years after the last treatment round) for previously untreated hyperendemic scenarios (baseline 70% mf prevalence) versus number of years of annual (squares) or biannual (six-monthly) (circles) community-directed treatment with ivermectin (CDTI, red markers and lines) or moxidectin (CDTM, blue markers and lines) assuming: (*a*) minimal coverage (65% treatment coverage of total population and 5% systematic non-adherence); (*b*) enhanced coverage (80% treatment coverage of total population and 1% systematic non-adherence). CDTI and CDTM are preceded by the letter ‘a’ for annual or ‘b’ for biannual treatment. The grey horizontal line indicates 90% elimination probability. (Online version in colour.)

Simulation results show that CDTM leads to higher elimination probabilities than CDTI when given with equal treatment frequency, coverage/adherence and treatment duration (figures 1–3). The proportion of simulations indicating EoT is highest with bCDTM, followed by bCDTI and aCDTM, with a large reduction in probability of EoT for aCDTI, but the relative impact of the ATS under consideration depends on initial endemicity.

### (a) Hypoendemic settings

In the treatment-naive hypoendemic scenarios (with no previous history of treatment), both CDTI and CDTM can help achieve EoT (figure 1). For example, with 12 years of aCDTI with minimal coverage, EoT is achieved in around 68% of the hypoendemic simulations, with this figure rising to 98% with aCDTM, and to 100% with bCDTI and bCDTM (figure 1*a*). To achieve 90% elimination probability, aCDTI would require 15 years with minimal coverage, or 11–12 years with enhanced coverage, but bCDTI would reduce programme duration by approximately 40% (to 7–8 years) irrespective of coverage, as minimal and enhanced coverage generate very similar results (figure 1*a,b*). For aCDTM, 90% elimination is reached in 11 years with minimal or 9 years with enhanced coverage, reducing programme duration by approximately a quarter (27%) or by 40%, respectively, compared with aCDTI with minimal coverage. Adopting a six-monthly strategy, bCDTM achieves 90% elimination within 7 years with minimal or 6 years with enhanced coverage, i.e. 8 years earlier than aCDTI with minimal coverage and 4–5 years earlier than aCDTM with minimal or enhanced coverage. The probabilities of elimination achieved with bCDTI and bCDTM are similar for the two coverage levels simulated.

### (b) Mesoendemic settings

For the treatment-naive mesoendemic scenarios, and following 20 years of treatment with minimal coverage, aCDTI is predicted to be capable of eliminating transmission in around 18% of the simulations, this value rising to 90% for aCDTM, and to 100% for bCDTI and bCDTM (figure 2*a*). aCDTI and aCDTM with minimal coverage can achieve EoT with 90% probability after 32 and 20 years, respectively (nearly a 40% reduction in programme duration with aCDTM). Increasing the treatment frequency to biannual, bCDTI and bCDTM with minimal coverage reduce the number of years to reach 90% EoT further to 15 and 11 years, respectively (an approximately 50 and 70% reduction in programme duration compared with aCDTI with minimal coverage) (figure 2*a*). Increasing coverage to 80% results in aCDTI leading to 90% probability of EoT in 21–22 years, whereas aCDTM and bCDTI would reduce the number of years required to achieve this by 30 and 50%, i.e. to 15 and 11 years, respectively (figure 2*b*). The maximum reduction in programme duration is achieved by bCDTM with enhanced coverage, which would require 9–10 years to achieve 90% probability of EoT, more than halving programme duration compared with aCDTI at the same (enhanced) coverage (figure 2*b*).

### (c) Hyperendemic settings

For previously untreated hyperendemic (70% mf prevalence) scenarios, model predictions indicate that aCDTI is clearly insufficient, with low probabilities of elimination regardless of coverage (figure 3*a,b*). In this case, bCDTM is the only treatment strategy predicted to reach 90% EoT probability with programme durations of 20 years at minimal coverage (figure 3*a*). With enhanced coverage, this would be achieved in 15–16 years of bCDTM (figure 3*b*). This contrasts with 40 or 22 years of bCDTI at minimal and enhanced coverage, respectively, and with 30 years of aCDTM at enhanced coverage (figure 3*a*,*b*). Twenty years of bCDTI are predicted to lead to 19% probability of elimination with minimal coverage, this value increasing to 85% with enhanced coverage. By contrast, 20 years would lead to 90% or nearly 100% probability of EoT with bCDTM, with minimal or enhanced coverage, respectively (figure 3*a*,*b*).

To further illustrate the contrasting impact of CDTI or CDTM in treatment-naive hyperendemic settings, electronic supplementary material, figure S4 shows the range of vector biting rates for which EPIONCHO-IBM predicts that EoT is achieved or not achieved after 40 years of MDA with enhanced coverage. While bCDTI and aCDTM improve elimination prospects compared with aCDTI, bCDTM greatly expands the range of ABR values for which EoT is predicted to be possible.

### (d) Settings with previous history of community-directed treatment with ivermectin

As most endemic areas have received aCDTI for many years, targeting onchocerciasis EPHP with 65% coverage of total population, we also simulated the impact of adopting an ATS following 20 years of aCDTI at minimal coverage. Electronic supplementary material, figure S5 presents the results for mesoendemic settings. Switching to bCDTM with enhanced coverage would only require 7–8 additional years of treatment to reach 90% EoT. Electronic supplementary material, figure S6 illustrates the substantial decrease in microfilarial load that follows the adoption of CDTM as an ATS in hyperendemic settings, particularly when implementing bCDTM at enhanced coverage.

### (e) Sensitivity analysis

We have thus far assumed that both ivermectin and moxidectin irreversibly decrease adult worm fecundity (sterilizing effect) by approximately 35% following each treatment round [3]. As described in Methods, we explored the effect of decreasing the cumulative effect due to ivermectin by half (to 17.5%) while keeping that of moxidectin at 35%, per dose, in simulations assuming minimal coverage. Results indicate that the parameter representing the magnitude of the assumed cumulative sterilizing effect is highly influential on model outputs (electronic supplementary material, figures S7–S9). For 30 and 50% mf prevalence settings, and when assuming that the effect of ivermectin is half that of moxidectin, aCDTM and bCDTI are approximately equivalent in their impact on achieving EoT (electronic supplementary material, figures S7*b* and S8*b*). aCDTM and bCDTI are also very similar for the high-transmission setting (70% mf prevalence) (electronic supplementary material, figure S9*b*), but neither can achieve EoT within 40 years of treatment. For this high-endemicity level, bCDTM is the only treatment strategy that can achieve EoT within a reasonable time frame (reaching a 90% elimination probability in 20 years, half the number of years compared with bCDTI; electronic supplementary material, figure S8*a*), in agreement with the results for bCDTM presented in figure 3*a*.

## 4. Discussion

Both the Phase II and III clinical trials showed that a single 8 mg dose of moxidectin is superior to a single 150 µg kg^−1^ dose of ivermectin at reducing and maintaining low skin microfilarial loads following treatment [26–28]. The EPIONCHO-IBM model can closely match the post-treatment microfilarial dynamics of *O. volvulus* observed in the 8 mg moxidectin treatment groups in these clinical trials using the existing functional forms that were derived for the microfilaricidal and embryostatic effects of single-dose ivermectin [2] and re-parameterized for single-dose moxidectin, with the above-mentioned doses [44]. Consistent with previous work [44], the modelling we present here predicts that moxidectin's stronger and more prolonged effect on skin microfilarial levels in individuals would translate into aCDTM yielding higher elimination probabilities and shorter times to achieve 90% probability of EoT compared with aCDTI when other factors (endemicity, treatment coverage and systematic non-adherence, duration of treatment and treatment history) are identical. In all scenarios, bCDTM was the strategy that reduced programme duration (number of years required to achieve EoT with 90% probability) the most.

Our modelling results indicate that the highest probabilities of elimination would be achieved by bCDTM, followed by bCDTI and aCDTM, with an appreciable drop in such probabilities for aCDTI for a given number of years of treatment when assuming that the cumulative effect of ivermectin and moxidectin on microfilarial productivity by adult female worms is the same, at 35% irreversible reduction, per dose, for both drugs. These results are somewhat different from those presented by Turner *et al*. [44], who used an earlier, deterministic version of EPIONCHO and concluded that aCDTM was approximately equivalent to bCDTI. In that study, programme duration was measured as the number of years of treatment necessary to achieve the provisional operational thresholds for treatment interruption followed by surveillance (pOTTIS)—based on microfilarial prevalence thresholds [51]. Therefore, the work presented in Turner *et al*. [44] did not calculate probabilities of elimination but, rather, treatment durations to reach a fixed mf prevalence applied to all endemicity levels, regardless of their intensity of transmission (see [47] for a discussion of this). Also, Turner *et al*. [44] investigated the impact of assuming different values of the, per-dose, irreversible sterilizing effect of treatment (ranging from 1 to 30%), but not the 35% used in the present work. Interestingly, when the assumed effect was at its lowest (1%), bCDTI required slightly more years than aCDTM to reach the pOTTIS, with this trend reversing for the largest assumed value (30%), in the direction of the results presented here.

The 35% value estimated in Plaisier *et al*. [3] was based on a modelling analysis of microfilarial load data obtained over five annual treatment rounds in an early community trial of ivermectin in Ghana [50]. Intuitively, the greater the magnitude of the, per-dose, irreversible effect on female worm fecundity, the larger the impact of six-monthly treatment would be. However, no evidence of a cumulative effect was found in another modelling study [54] of microfilarial data from hyperendemic communities in Guatemala over five biannual treatments [55]. Likewise, an analysis of microfilarial densities after ivermectin treatment in treatment-naive and multiply treated populations from Cameroon did not support the operation of a strong cumulative effect of repeated treatments on the fecundity of female worms [56]. Therefore, to shed light on this important assumption, it is crucial that empirical, parasitological evidence be obtained from the ongoing field trial comparing the efficacy of ivermectin and moxidectin at annual and biannual frequencies over a number of treatment rounds [57]. Our modelling study also assumes that the efficacies of the microfilaricidal and embryostatic effects of ivermectin and moxidectin remain the same over the entire programme duration, i.e. that prolonged MDA does not lead to changes in the susceptibility of the parasite population to either drug.

The results of our sensitivity analysis highlight the need to better understand any differences that may exist between the irreversible sterilizing effect of ivermectin and moxidectin, and/or any other anti-macrofilarial effect. A greater magnitude (14 times larger) of the embryostatic effect of moxidectin compared with that of ivermectin (with a rate of decay four times smaller) was estimated in Turner *et al*. [44] when fitting the functions presented in Basáñez *et al*. [2] to the Phase II clinical trial data of [26] (electronic supplementary material, table S1). Data suggesting that it is plausible to hypothesize that the sterilizing effect of repeat moxidectin treatment may be at least as large (if not larger) than that of repeat ivermectin treatment include: (i) the longer half-life of moxidectin (20–30 days) compared with that of ivermectin (approximately 1 day) [40,58]; (ii) the stronger effect on skin mf levels of 2 mg moxidectin compared with ivermectin at 6 and 12 months following treatment [26]; (iii) the presence of very few individuals with detectable microfilaridermia six months post-treatment in the 8 mg moxidectin group of the Phase II and III trials [26–28]; and (iv) the nine times greater proportion of individuals without detectable microfilaridermia 12 months post-treatment in the 8 mg moxidectin arm of the Phase III trial compared with the ivermectin arm [27]. The longer half-life of moxidectin may also result in any cumulative effects on female worm sterilization being more pronounced after six-monthly than yearly treatment. Modelling using data from an ongoing trial [57] will be important to evaluate whether the parameters derived from the single-dose studies appropriately capture the microfilaricidal and embryostatic effects of ivermectin and moxidectin during annual or biannual repeat treatment, and to gain insight into the sterilizing effects of both drugs.

Our modelling did not consider macrofilaricidal effects for either drug. A modelling study [5] of the adult worm data obtained in a 3-year field trial conducted in Cameroon [59] showed that multiple doses of ivermectin may have a partial macrofilaricidal effect even at annual frequency, which increased at quarterly (three-monthly) frequency, whereas the permanent sterilizing effect was more modest [5]. Moxidectin may have different or more pronounced effects that account for the longer suppression of microfilaridermia, which are not modelled here, potentially including prophylactic effects against incoming L3 larvae [6,60]. Again, longer-term efficacy studies as well as experimental studies in animal models will be necessary to understand any of these.

Notwithstanding the overall result that bCDTM represents the strategy that accelerates onchocerciasis EoT the most in all modelled scenarios, the question arises as to the epidemiological settings where adoption of CDTM would add the greatest value. In hypoendemic areas, bCDTI could be used to reach elimination goals even at minimal coverage (figure 1*a*), while in mesoendemic settings, if bCDTI encountered operational challenges, aCDTM at enhanced coverage would be a suitable strategy (figure 2*b*). In these areas, and provided six-monthly treatment was feasible, bCDTI with enhanced coverage (figure 2*b*) or bCDTM at minimal coverage (figure 2*a*) would greatly accelerate progress towards elimination. In hyperendemic settings, only bCDTM, in particular with enhanced coverage, can improve elimination prospects (figure 3), and lead to EoT in the largest range of vector biting rates (electronic supplementary material, figure S4). This is consistent with the notion that EoT becomes increasingly difficult as the level of baseline endemicity increases, as these levels are associated with greater values of ABR (and thus of the basic reproduction number, *R*_0_, of the infection) [61]. If prolonged aCDTI at minimal coverage has proved ineffective, switching to bCDTM with enhanced coverage would decrease microfilarial loads between treatment rounds dramatically, thus reducing vector infection rates and consequently reducing transmission and accelerating progress towards EoT (e.g. electronic supplementary material, figures S5 and S6 for mesoendemic and hyperendemic settings, respectively).

## 5. Limitations

Beyond the limitations inherent in the modelling assumptions discussed above, our study has the following limitations:

*Vector biting rates.* Our study assumed that the ABRs used to obtain baseline microfilarial prevalence values would remain the same throughout the entire duration of the programme. However, this may not be the case owing to environmental, ecological and anthropogenic change. In the absence of robust data on secular trends in vector biting rates, it is difficult to incorporate these into the model. Declines in vector density could contribute to reaching EoT earlier than our projections indicate, and to minimizing resurgence. In fact, studies conducted in a number of endemic areas where vector control had not been implemented have reported absence, near-absence or substantial declines of blackfly vectors when epidemiological and entomological investigations were carried out during monitoring and evaluation or stop-MDA surveys, likely owing to environmental change. Such declines have likely contributed to EoT [11,12,14].

*Exposure heterogeneity.* Throughout our simulations (30 to 70% baseline mf prevalence), we assumed the default values of exposure heterogeneity and density dependence in parasite establishment within humans estimated in Hamley *et al*. [45]. However, higher mf prevalence values would be better captured by weaker exposure heterogeneity and density dependence, and this could lead to higher probabilities of EoT (see fig. 5 of [45]). Therefore, simulations could be conducted by way of sensitivity analysis using different sets of exposure heterogeneity and density dependence parameters to reflect the current uncertainty surrounding these modelling assumptions.

## 6. Conclusion, recommendations and considerations for the adoption of moxidectin mass drug administration as an alternative treatment strategy

Our modelling results support the potential of moxidectin as an ATS to accelerate progress towards EoT. The safety profile of moxidectin established in Phase I (uninfected, healthy volunteers) and Phase II and III trials suggests that moxidectin-based ATS could use existing programmatic structures for community-directed treatment. The extent to which this potential can be fully realized and considered by WHO in decisions to include moxidectin in treatment guidelines for the elimination of onchocerciasis will depend on a number of factors:

**Regulatory approval for treatment of children under 12 years of age.** The US FDA has approved moxidectin for the treatment of onchocerciasis in those aged greater than or equal to 12 years [40,42]. Modelling results (data not shown) indicate that treating the population aged greater than or equal to 5 years with moxidectin would be a more effective strategy than treating the 5- to 11-years olds with ivermectin and those aged greater than or equal to 12 years with moxidectin [62]. Moreover, a ‘two-drug strategy’ would greatly complicate the logistics of community-directed treatment and hinder community mobilization, because, among other considerations, ivermectin (but not moxidectin), is dosed by height [40]. The pharmacokinetic and safety study to identify moxidectin doses for children aged 4–11 years that result in exposures comparable to those obtained in adults treated with an 8 mg dose has been completed and final analysis is ongoing [46]. Medicines Development for Global Health (MDGH, https://www.medicinesdevelopment.com/uk) will submit the data to, and identify in consultation with the US FDA, any additional information required to support expanding US FDA approval to this age group. MDGH will also provide study data and conclusions to WHO and to any regulatory agency reviewing applications for use of moxidectin in children in pilot field projects.

**Acceptability of moxidectin to local healthcare workers, community drug distributors and community members.** While ivermectin is generally well accepted by endemic communities for delivery by community drug distributors (CDDs), the acceptability of moxidectin by CDDs and community members needs to be ascertained. Factors affecting the success of community-directed treatment in general and CDTI implementation in particular have been investigated, ranging from community engagement and participation to the experiences of CDDs and health workers and their interaction with the health system [63,64]. These factors, among many others, including drug acceptability, strongly impact treatment coverage and adherence levels. One determinant of acceptability is the drug safety profile. Data available to date indicate no significant differences between moxidectin and ivermectin regarding safety. Further safety data will become available [57,65], with the latter protocols comparing the safety of single doses of moxidectin and ivermectin in individuals with and without parasitological evidence of *O. volvulus* infection. Pilot field projects will be needed to evaluate other determinants of acceptability.

**Cost and cost-effectiveness of moxidectin mass drug administration.** Ivermectin is donated by Merck & Co. through the Mectizan Donation Program (MDP), but the financial basis for moxidectin provision to countries still needs to be delineated. As a not-for-profit organization, MDGH operates within a different financial context compared with Merck & Co. and is working to fulfil its commitment to WHO in the agreement under which WHO licensed all data (including the results of the Phase II and III trials) to MDGH to register moxidectin and make it available in line with the not-for-profit principles in MDGH's constitution. MDGH is actively exploring opportunities to support donation. Cost and cost-effectiveness analysis for different moxidectin use scenarios [44] will need to be conducted to inform country decisions.

**Optimization of treatment coverage/adherence.** Increasing treatment coverage from the minimum of 65% of total population (here assumed to be accompanied by 5% systematic non-adherence) for EPHP to 80% (with assumed 1% of systematic non-adherence) will be crucial for achieving EoT with CDTI or CDTM. Maximizing treatment coverage (with reliable documentation), and understanding, addressing and minimizing the many (locale-specific) factors that result in systematic non-adherence, have represented a challenge from the very beginning of CDTI. These challenges have been highlighted in all APOC programme reviews [66] as well as in reports of APOC's Technical Consultative Committee meetings and APOC progress reports [67]. The portal of the Expanded Special Project for Elimination of Neglected Tropical Diseases (ESPEN, https://espen.afro.who.int/) collates and regularly updates country data on reported treatment coverage over implementation/evaluation units and years. To improve modelling, studies quantifying compliance patterns over consecutive treatment rounds, such as those conducted for soil-transmitted helminthiases in Kenya and Ethiopia [68,69], are needed to complement prior studies into compliance with CDTI [30,32,70–72].

**Feasibility of implementing biannual treatment where necessary.** This is particularly important in areas of meso- to hyperendemicity (in those that have received no treatment or very few treatment rounds as well as in those with a long history of aCDTI but with less than desired progress towards EoT) and may be worth considering elsewhere (e.g. cross-border transmission areas, where progress towards elimination in one country lags behind that achieved in a neighbouring country, risking re-introduction of infection by cross-border population and vector movement). The feasibility of implementing six-monthly treatment may differ between countries, endemic areas, populations to be treated (e.g. migratory versus sedentary) and over time (e.g. during periods of civil unrest, disease outbreaks).

**Feasibility of optimizing treatment time according to transmission seasonality.** Optimizing the time of treatment relative to seasonal transmission has been, together with optimizing treatment coverage, a key recommendation for maximizing CDTI impact since the commencement of CDTI. This requires consideration particularly in areas where transmission is highly seasonal, to ensure that skin mf levels have decreased to their lowest when vector biting rates are highest and that when mf start repopulating the skin there are no or very few vectors around to ingest such mf [20,73]. The longer duration of microfilaridermia suppression due to moxidectin makes the impact of CDTM less dependent on optimal timing of treatment than CDTI [44].

## 7. Future research avenues

Both the Phase II [26] and Phase III [27] clinical trial data indicated that the magnitude of inter-individual variation in responses to treatment was greater for ivermectin than for moxidectin in a treatment-naive population following single-dose treatment (see also electronic supplementary material, figure S1), suggesting that sub-optimal responses may occur more frequently with ivermectin treatment, and be present prior to prolonged MDA, as previously shown [74]. Subsequent analysis of the Phase III data [28] also indicated that the percentage of individuals with skin mf density at 12 months post-treatment greater than 40% of that at pre-treatment for ivermectin versus moxidectin differed between the localities from which trial participants were recruited (28 versus 4% in Nkwanta district, Ghana; 24 versus 0.3% in Ituri and 12 versus 0% in Nord Kivu, Democratic Republic of Congo; 11 versus 2% in Lofa County, Liberia), suggesting that (potentially genetic) differences between parasite populations in different geographical areas play a role in treatment responses [17] and progress towards EoT.

If individuals responding well or less well to ivermectin (or moxidectin) were to continue doing so over subsequent treatment rounds, this could have implications for elimination prospects, with preliminary modelling data (not shown) indicating that this systematic variation would confer an additional advantage to moxidectin over ivermectin. If inter-individual variation in treatment responses were random, the results would be very similar to those presented here [62]. Therefore, an important future research question is to elucidate the patterns of intra- and inter-individual variation in treatment responses over multiple rounds of ivermectin or moxidectin in field trials to complement the data that will become available [57].

Likewise, the potential prophylactic effect of ivermectin and moxidectin should be investigated. Individuals treated three-monthly with ivermectin had fewer new onchocercal nodules than those treated annually [6]. *In vitro* studies with L3 and L4 *O. volvulus* larvae found that both ivermectin and moxidectin inhibited moulting of the former and motility of the latter, with moxidectin being more effective than ivermectin [60]. In cattle, both monthly and three-monthly treatment with ivermectin or moxidectin prevented establishment of adult *Onchocerca ochengi* worms in infection-naive cows compared with untreated, transmission-exposed animals [75]. Preliminary modelling data (not shown) suggest that inclusion of moxidectin's prophylactic effect into EPIONCHO-IBM appreciably increases EoT probabilities, with 10% more simulations achieving elimination for both aCDTM and bCDTM [62]. Therefore, the complex interplay between transmission intensity, exposure, parasite establishment, immune system responses and potential macrofilaricidal and prophylactic effects of treatment with ivermectin or moxidectin in human communities warrants the refinement of onchocerciasis transmission models that incorporate such effects and allow investigation of their (likely) highly nonlinear interactions to improve elimination projections towards 2030 and beyond.

## Data Availability

The model code is available from the GitHub repository: https://github.com/mrc-ide/EPIONCHO.IBM. All information used for the analyses is contained in the figures and the electronic supplementary material [76].
